# Hyperactivation of platelet‐derived growth factor signalling contributes to arrhythmogenesis in Brugada syndrome

**DOI:** 10.1002/ctm2.715

**Published:** 2022-02-20

**Authors:** Hongkun Wang, Lizhen Xu, Shuai Han, Xiaochen Wang, Hao Wang, Jingjun Zhou, Jiaxi Shen, Zongkuai Yang, Luyang Yu, Zhouqing Huang, Tingyu Gong, Ming Qi, Fan Yang, Ping Liang

**Affiliations:** ^1^ Key Laboratory of combined Multi‐organ Transplantation, Ministry of Public Health, the First Affiliated Hospital Zhejiang University Hangzhou China; ^2^ Institute of Translational Medicine Zhejiang University Hangzhou China; ^3^ Department of Biophysics, and Kidney Disease Center of the First Affiliated Hospital Zhejiang University School of Medicine Hangzhou China; ^4^ Department of Obstetrics, Zhejiang Provincial People's Hospital People's Hospital of Hangzhou Medical College Hangzhou China; ^5^ Department of Prenatal Diagnosis (Screening) Center Hangzhou Women's Hospital (Hangzhou Maternity and Child Health Care Hospital) Hangzhou China; ^6^ College of Life Sciences Zhejiang University Hangzhou China; ^7^ Department of Cardiology the First Affiliated Hospital of Wenzhou Medical University Wenzhou China; ^8^ Department of Cell Biology and Medical Genetics Zhejiang University School of Medicine Hangzhou China

Dear Editor,

We performed a comprehensive study to assess the pathogenicity of a novel transient receptor potential melastatin 4 (TRPM4) mutation and to pinpoint underlying molecular mechanisms using an induced pluripotent stem cell‐derived cardiomyocyte (iPSC‐CM) model. Patient‐specific iPSC‐CMs exhibited arrhythmic phenotype manifesting delayed afterdepolarizations (DADs) and paroxysmal cellular flutter (PCF), which were rescued by correction of the causal mutation. T262M conferred impaired TRPM4 channel function by enhanced ubiquitination for protein degradation via the lysosomal pathway, resulting in abnormal Ca^2+^ cycling and elevated diastolic intracellular Ca^2+^ ([Ca^2+^]_i_). Mechanistically, hyperactivation of platelet‐derived growth factor receptor β (PDGFRB) signalling derived arrhythmic events in diseased iPSC‐CMs. On the contrary, pharmacological and genetic inhibition of PDGFRB effectively restored diastolic [Ca^2+^]_i_ and rescued the arrhythmic phenotype in T262M myocytes.

Brugada syndrome (BrS) is an inherited arrhythmogenic disease featured by a high risk of sudden cardiac death.^1^ TRPM4 mutations have been reported to be associated with BrS,^2^, ^3^, ^4^ whereas the underlying mechanisms remain unknown.

In this study, we recruited a 21‐year‐old male patient who is asymptomatic and no abnormality was found by echocardiography. However, his electrocardiogram showed a characteristic type 2 BrS pattern, with ST‐segment morphology representing saddleback‐type elevation in lead V2 (Figure S1A). In addition, we further monitored the electrocardiogram in lead V1 and V2 from one or two intercostal spaces higher than the standard position (Figure S1B). The genetic screening revealed a single missense mutation (c.785C > T, p.T262M) in *TRPM4* (Figure S1C–E). The mutation, located at the N terminus of the TRPM4 channel, is highly conserved among spices and is graded as a variant of uncertain significance (Figure S1F,G). Our healthy control subject was a 21‐year‐old female. Skin fibroblasts were reprogrammed using nonintegrated Sendai virus and iPSCs were successfully generated and characterized (Figures S1H–L, S2 and S3). Genetic sequencing confirmed that T262M mutation was present in patient iPSCs but not in controls (Figure S1M). The iPSC‐CMs were subsequently generated by a small molecule‐based monolayer differentiation protocol (Figures S4 and S5).

Single‐cell patch clamp recordings revealed a uniform and rhythmic action potential (AP) profile in control iPSC‐CMs (Figure 1A). However, arrhythmic waveforms were seen in a large proportion of patient iPSC‐CMs, manifesting two distinct phenotypes: the more common phenotype was DAD; the other rare but more severe phenotype was PCF (Figure 1B,C,G). Moreover, we observed significantly increased peak interval variability and shortened AP duration (APD) in patient iPSC‐CMs (Figure 1H,I and Table S1). We next generated isogenic control lines by CRISPR/Cas9‐mediated genome editing technology (Figure S2 and S6). The gene‐corrected (GC) iPSC‐CMs showed a dramatic reduction of arrhythmic incidence and resembled the AP profile of controls (Figure 1D,G–I, Figures S4, S5 and Table S1). TRPM4 knockout (KO) iPSC lines were also generated by CRISPR/Cas9 (Figures S2, S7 and S8). KO iPSC‐CMs recapitulated abnormal AP phenotype of patient iPSC‐CMs (Figure 1E–I, Figures S4, S5 and Table S1). Taken together, these results demonstrate that TRPM4 T262M is a pathologic mutation that causes the arrhythmic phenotype.

**FIGURE 1:**
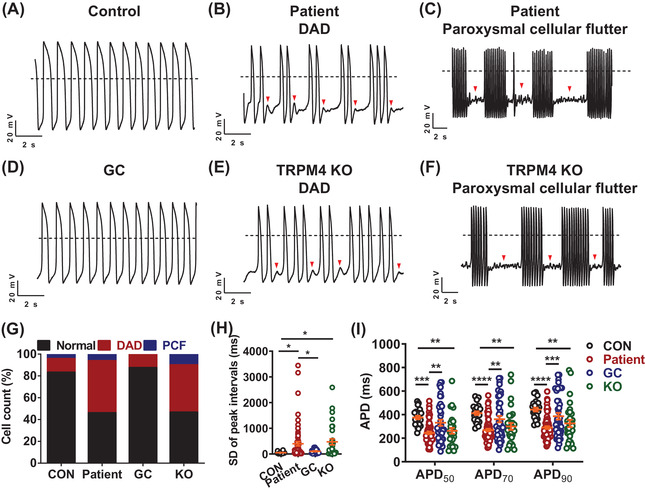
TRPM4 T262M leads to cardiac arrhythmias in iPSC‐CMs. (A–F) Representative action potential tracings recorded by single‐cell patch clamp from control, patient, gene‐corrected (GC) and TRPM4 knockout (KO) ventricular‐like myocytes. Dashed lines indicate 0 mV. (G) Bar graph to compare the percentage of cells with arrhythmias between control (CON), patient, GC and KO iPSC‐CMs. Control: 16.7%; Patient: 53.8%; GC: 12.5%; KO: 53.3%. *n* = 24–65 in two lines. (H) Scatter dot plot to compare SD of peak intervals between control, patient, GC and KO iPSC‐CMs by one‐way analysis of variance (ANOVA) (Tukey method). *n* = 24–65 in two lines. ^*^ *p *< .05. (I) Scatter dot plot to compare action potential durations (APDs) between control, patient, GC and KO iPSC‐CMs by two‐way ANOVA (Tukey method). *n* = 24–65 in two lines. ^**^ *p *< .01, ^***^ *p *< .001 and ^****^ *p *< .0001

To assess if T262M gave rise to TRPM4 channel dysfunction, we next performed patch clamp on human embryonic kidney 293T cells transiently expressing TRPM4 (Figure 2A). The current density was significantly reduced in T262M channels as compared to wildtype (WT) (Figure 2B,C). Through molecular modelling, we observed no significant change of TRPM4 channel structure affected by T262M (Figure S9). Biotinylation assay revealed that total and surface expression levels of TRPM4 protein were significantly decreased in T262M channels, whereas the ratio of surface‐to‐total expression levels remained unchanged (Figure 2D–G). Consistently, the endogenous TRPM4 protein expression was markedly decreased in patient iPSC‐CMs (Figure 2H,I). Given that the TRPM4 channel is activated by [Ca^2+^]_i_,^5^ we sought to investigate if T262M affected TRPM4 channel activation upon [Ca^2+^]_i_ stimulation. Patch clamp recordings were performed in inside‐out mode, allowing the cytosolic side of the patch perfused by escalated [Ca^2+^]_i_. We observed significantly reduced [Ca^2+^]_i_‐activated TRPM4 current density and right‐shifted current‐[Ca^2+^]_i_ curve in T262M channels, suggesting a weakened sensitivity to [Ca^2+^]_i_ (Figure 2J–M). Moreover, we observed a markedly elevated level of ubiquitinated TRPM4 protein in T262M channels using the ubiquitin antibody P4D1 (Figure S10A,B). Inhibition of lysosome pathway by chloroquine effectively rescued T262M‐induced down‐regulation of TRPM4 protein and reduction of TRPM4 currents (Figure S10C–I). Collectively, these results indicate that T262M confers impaired TRPM4 channel function by enhanced ubiquitination for protein degradation via the lysosomal pathway.

**FIGURE 2:**
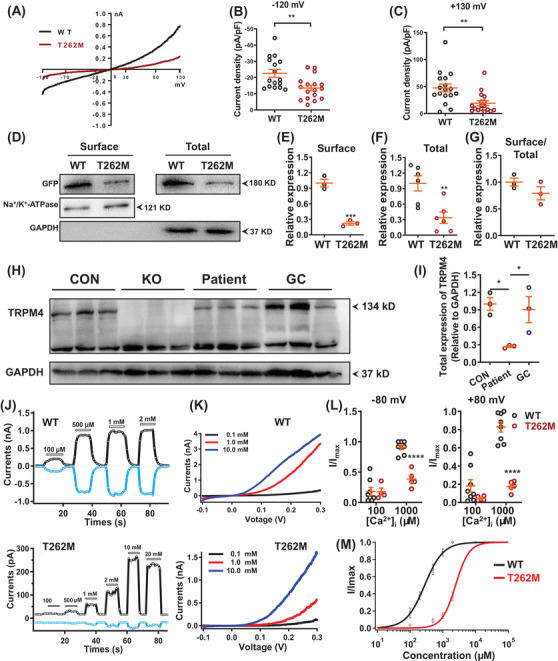
T262M confers impaired TRPM4 channel function. (A) Current‐voltage curves (IV curves) of whole‐cell TRPM4 currents recorded from human embryonic kidney 293T cells (HEK293T cells) overexpressing WT and T262M channels labelled with green fluorescent protein (GFP) at C terminus. Successfully transfected cells, indicated by green fluorescence, were patch‐clamp recorded for macroscopic currents using a voltage ramp protocol for 250 ms from –120 mV to +130 mV. (B, C) Scatter dot plots to compare the peak TRPM4 current density at ‐120 mV and +130 mV between WT and T262M by unpaired two‐tailed Student's *t*‐test (at –120 mV: WT = 22.6 ± 2.4 pA/pF, T262M = 13.5 ± 1.7 pA/pF; at +130 mV: WT = 47.4 ± 7.3 pA/pF, T262M = 19.1 ± 4.5 pA/pF). *n* = 16–17 patches. ^**^ *p *< .01. (D) Western blot analysis of surface and total protein expression of TRPM4 in HEK293T cells overexpressing WT and T262M. (E–G) Scatter dot plots to compare the surface expression of TRPM4, total expression of TRPM4, and TRPM4 surface expression/TRPM4 total expression (surface/total) between WT and T262M by unpaired two‐tailed Student's t‐test, respectively. *n* = 3–6 independent experiments. ^**^ *p *< .01 and ^***^ *p *< .001. (H) Western blot analysis of total protein expression of TRPM4 in control, KO, patient and GC iPSC‐CMs. (I) Scatter dot plot to compare total protein expression of TRPM4 in control, patient and GC iPSC‐CMs by one‐way analysis of variance (ANOVA) (Tukey method). *n* = 3 independent experiments. ^*^ *p *< .05. (J) Representative TRPM4 current tracings of WT and T262M by inside‐out patch‐clamp recordings at +80 mV (black) and –80 mV (blue). (K) Representative IV curves of TRPM4 currents in WT and T262M by inside‐out patch clamp recordings. Three different Ca^2+^ concentrations were used including 0.1, 1.0 and 10.0 mM. In WT channels, currents were robustly obtained at 100 μM [Ca^2+^]_i_ and the [Ca^2+^]_i_‐activated currents saturated when the [Ca^2+^]_i_ concentration reached 500 μM. In contrast, T262M channels gave rise to detectable currents upon perfusion of 500 μM [Ca^2+^]_i_, which appeared significantly larger at 1000 μM [Ca^2+^]_i_ and saturated at 10000 μM [Ca^2+^]_i_. (L) Currents normalized to the response to 100 and 1000 μM Ca^2+^ at –80 mV or +80 mV as in the experiments in Figure 2J by two‐way ANOVA (Tukey method). *n* = 4–8 patches. ^****^ *p *< .0001. (M) The concentration‐response curves of WT and mutant T262M with Ca^2+^ concentrations were measured with inside‐out patch‐clamp recordings. *n* = 3–5 patches

Alterations in Ca^2+^ cycling are a common trigger of cardiac arrhythmias.^6^ More attention has been attracted to the roles of Ca^2+^ signalling in arrhythmogenic mechanisms of BrS.^7^ To test whether T262M affects Ca^2+^ homeostasis, Ca^2+^ imaging was performed using fura‐2 AM dye to ratiometrically record Ca^2+^ transients in iPSC‐CMs (Figure S11A–C). Patient iPSC‐CMs exhibited “arrhythmia‐like” irregular transients, dysregulation of Ca^2+^ cycling and elevation of diastolic [Ca^2+^]_i_ (Figure S11D–L and Table S2). Interestingly, L‐type Ca^2+^ current density was markedly increased in T262M iPSC‐CMs, and treatment of verapamil in T262M iPSC‐CMs drastically ameliorated the incidence of irregular transients (Figure S11M–T). These results demonstrate that disrupted Ca^2+^ homeostasis is associated with arrhythmic phenotype caused by T262M.

To understand the molecular mechanisms of how TRPM4 T262M causes arrhythmic phenotype, we performed genome‐wide RNA sequencing by comparing control, KO, patient and GC iPSC‐CMs (Figure 3A–C). Gene ontology analysis revealed that differentially expressed genes were enriched in ion channel‐, Ca^2+^ signalling‐, cardiac action potential and conduction, and PDGF signalling‐related terms (Figure 3D). Interestingly, we found greatly enhanced PDGFRB expression in KO or patient iPSC‐CMs (Figure 3D–H). Previous studies have reported that PDGFRB signalling regulates cardiomyocyte proliferation and myocardial regeneration, and over‐activation of PDGFRB signalling is closely associated with atrial fibrillation and dilated cardiomyopathy.^8^, ^9^, ^10^ Notably, treatment of PDGFRB inhibitors or knockdown of PDGFRB expression in patient iPSC‐CMs largely reduced PDGFRB protein expression, greatly attenuated proarrhythmic activities, and restored elevated diastolic [Ca^2+^]_i_ (Figure 4). Altogether, these results suggest that hyperactivation of PDGFRB signalling contributes to arrhythmogenesis in TRPM4‐related BrS.

**FIGURE 3:**
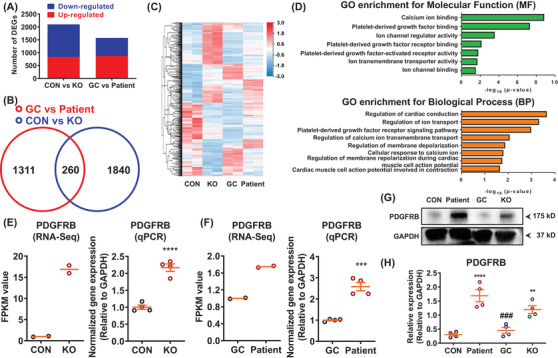
RNA sequencing analysis reveals abnormal activation of platelet‐derived growth factor signalling in patient iPSC‐CMs. (A) Bar graph to compare the number of differentially expressed genes (DEGs) between control and KO, as well as GC and patient iPSC‐CMs. *n* = 2 independent samples. (B) Venn diagram to compare DEGs between control and KO, as well as GC and patient iPSC‐CMs. (C) Heatmap demonstrating the differential gene expression pattern between control and KO, as well as GC and patient iPSC‐CMs. By one set of RNA sequencing (RNA‐Seq) analysis using samples of GC and patient iPSC‐CMs (GC vs. patient), we observed that 1573 genes out of 18 805 total genes were differentially expressed in GC iPSC‐CMs (869 up‐regulated and 704 down‐regulated) as compared to patient iPSC‐CMs. By another set of RNA‐Seq analyses using samples of control and TRPM4 KO iPSC‐CMs (CON versus KO), 2100 DEGs were found in control iPSC‐CMs (834 up‐regulated and 1266 down‐regulated), when compared to KO iPSC‐CMs. A cross‐analysis of GC versus patient and CON versus KO identified 260 common DEGs, in which 95 genes were up‐regulated and 165 genes were down‐regulated, respectively. (D) Enriched gene ontology (GO) for molecular function (MF) and biological process (BP). DEGs were enriched in platelet‐derived growth factor (PDGF) signaling, including “PDGF binding”, “PDGF receptor binding”, “PDGF‐activated factor activity” and “PDGF receptor signaling pathway”. (E) Scatter dot plots to compare the mRNA expression of PDGFRB between control and KO iPSC‐CMs by RNA‐Seq (*n* = 2 independent samples) and qPCR (*n* = 4 independent experiments) by unpaired two‐tailed Student's *t*‐test. ^****^ *p *< .0001. (F) Scatter dot plots to compare the mRNA expression of PDGFRB between GC and patient iPSC‐CMs by RNA‐Seq (*n* = 2 independent samples) and qPCR (n = 4 independent experiments) by unpaired two‐tailed Student's *t*‐test. ^***^ *p *< .001. (G) Western blot analysis of total protein expression of PDGFRB in control, patient, GC and KO iPSC‐CMs. (H) Scatter dot plot to compare total protein expression of PDGFRB in different groups by one‐way analysis of variance (ANOVA) (Tukey method). *n* = 4 independent experiments. ^**^ *p *< .01 and ^****^ *p *< .0001, when compared to control iPSC‐CMs; ^###^ *p *< .001 when compared to patient iPSC‐CMs

**FIGURE 4:**
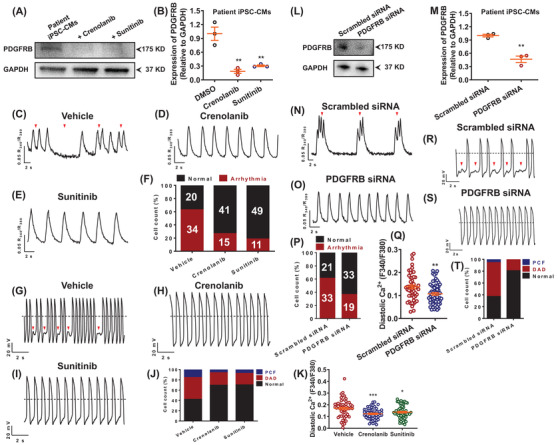
Restoration of diastolic [Ca^2+^]_i_ and rescue of arrhythmic phenotypes by inhibition of PDGFRB signalling. (A) Western blot analysis of total protein expression of PDGFRB in patient iPSC‐CMs treated with dimethyl sulfoxide (DMSO) and two PDGFRB inhibitors (crenolanib and sunitinib). (B) Scatter dot plot to compare total protein expression of PDGFRB in different groups by unpaired two‐tailed Student's *t*‐test. *n* = 3 independent experiments. ^**^ *p *< .01. (C–E) Representative Ca^2+^ transient tracings from patient iPSC‐CMs treated with DMSO, crenolanib or sunitinib. (F) Bar graph to compare the percentage of cells exhibiting regular and irregular Ca^2+^ transient pattern between different groups. *n* = 54–60 in two lines. The proarrhythmic activities were greatly attenuated when treated with crenolanib or sunitinib as evidenced by Ca^2+^ imaging. (G–I) Representative action potential tracings from patient iPSC‐CMs treated with DMSO, crenolanib or sunitinib. (J) Bar graph to compare the percentage of cells with arrhythmias between patient iPSC‐CMs treated with DMSO, crenolanib and sunitinib. *n* = 26–33 in two lines. The proarrhythmic activities were greatly attenuated when treated with crenolanib or sunitinib as evidenced by patch clamp recordings. (K) Scatter dot plot to compare diastolic [Ca^2+^]_i_ between different groups by unpaired two‐tailed Student's *t*‐test. The elevation of diastolic [Ca^2+^]_i_ in myocytes carrying TRPM4 T262M was restored by crenolanib or sunitinib treatment. *n* = 54 in two lines. ^*^ *p *< .05 and ^***^ *p *< .001. (L) Western blot analysis of total protein expression of PDGFRB in patient iPSC‐CMs treated with scrambled or PDGFRB siRNA. (M) Scatter dot plot to compare total protein expression of PDGFRB between the two groups by unpaired two‐tailed Student's *t*‐test. *n* = 3 independent experiments. ^**^ *p *< .01. (N, O) Representative Ca^2+^ transient tracings from patient iPSC‐CMs treated with scrambled or PDGFRB siRNA. (P) Bar graph to compare the percentage of cells exhibiting regular and irregular Ca^2+^ transient patterns between the two groups. *n* = 52–54 in two lines. (Q) Scatter dot plot to compare diastolic [Ca^2+^]_i_ between the two groups by unpaired two‐tailed Student's *t*‐test. *n* = 52–59 in two lines. ^**^ *p *< .01. (R, S) Representative action potential tracings from patient iPSC‐CMs treated with scrambled or PDGFRB siRNA. (T) Bar graph to compare the percentage of cells with arrhythmias between patient iPSC‐CMs treated with scrambled and PDGFRB siRNA. *n* = 36 in two lines

In conclusion, genome editing of iPSC‐CMs can offer a precision medicine approach for identifying pathogenic mutation of BrS in a dish. More importantly, our findings reveal novel molecular mechanisms and potential therapeutic targets of TRPM4‐related BrS (Figure S12).

## Supporting information

Supporting Information
