# Supplementary material for: Hyperactivation of platelet‐derived growth factor signalling contributes to arrhythmogenesis in Brugada syndrome
Source: Clin Transl Med. 2022 Feb 20;12(2):e715. doi: 10.1002/ctm2.715 (PMC8858633; doi:10.1002/ctm2.715)
Supplement: Supplementary file 1 — Supporting Information [file CTM2-12-e715-s001.pdf]

SUPPLEMENTAL MATERIALS AND METHODS

Patient recruitment

Skin biopsies were obtained from a patient with suspected Brugada syndrome. The study conformed to the principles in the Declaration of Helsinki. Consent form was signed by the patient, and this study was approved by the Ethics Committee of the First Affiliated Hospital, Zhejiang University School of Medicine (No. 2019-497).

Skin biopsies and maintenance of fibroblasts

Freshly isolated skin biopsies were rinsed with Dulbecco's Phosphate-Buffered Saline (DPBS) and transferred into a 1.5-ml tube. Tissue was minced in collagenase I (1 mg/ml in Dulbecco's modified Eagle medium (DMEM), Gibco) and allowed to digest for 6 hours at 37 ℃. Dissociated dermal fibroblasts were plated and maintained with DMEM containing 10% FBS (Gibco) and Penicillin Streptomycin (Gibco) at 37 ℃, 95% air and 5% CO_2 in a humidified incubator. All cells were used for reprogramming within 5 passages.

Generation of iPSC lines

Somatic reprogramming was used to iPSC lines from skin fibroblasts using CytoTune-iPS 2.0 Sendai Reprogramming Kit following the manufacturer's instructions (Life Technologies). At least two iPSC lines were generated from each individual and used for downstream investigations **(Table S3)**.

Culture and maintenance of iPSCs

The iPSCs were cultured in feeder-free mTeSR1 (STEMCELL Technologies) media on matrigel-coated (Corning) plates at 37 ℃ with 5% (vol/vol) CO_2. The media were

daily changed and cells were passaged every 3-4 days using Accutase (STEMCELL Technologies).

Generation of TRPM4 knockout iPSC lines by CRISPR/Cas9

The exon 2 of *TRPM4* was selected for guide RNA (gRNA) design and the gRNA was designed according to the CRISPR online design tool (http://crispr.mit.edu/). The sequences of a pair of oligos for targeting site were listed as followed: Forward, 5’-CACCGGTCAACTATGAACGTCGTGC-3’; Reverse, 5’-AAACGCACGACGTTCATAGTTGACC-3’. The oligos were annealed and ligated into linearized lentiCRISPRv2 vector for generating gRNA-expressing plasmid as previously described. 1×10^5 control iPSCs were seeded in 12-well plates (Corning) to 80% confluence. The recombinant plasmid was transfected into control iPSCs using Lipofectamin 3000 Transfection Reagent (Invitrogen) according to the manufacturer's instructions. The cells were placed in mTeSR1 supplemented with puromycin (Xiao You Biotechnology) at a concentration of 4 μg/ml. After 2-3 days, puromycin resistant clones were picked and then verified by genomic PCR and DNA sequencing (Sangon Biotech).

Genetic screening and analysis

Patient iPSCs were cultured with mTeSR1 media on Matrigel-coated 6-well plates, and obtained at 80-90% confluence for subsequent analysis. Genomic DNA was extracted using a commercial DNA isolation kit (Qiagen). Ploymerase chain reaction (PCR) was carried out on EasyCycler 96 (Analytik Jena). Single nucleotide polymorphism (SNP) loci within *TRPM4* gene (c.785C>T, p.T262M) was amplified

and analyzed by direct sequencing, and then confirmed by sub-cloning. The forward and reverse primer sequences for *TRPM4* are 5'-TTCTTTGTTTCTCTCCCCCT-3' and 5'-TTGAATTACTGGCCTCAAGCA-3', respectively.

Karyotyping

Chromosome preparation and karyotype analysis were performed according to standard protocols by the Department of Prenatal Diagnosis (Screening) Center of Hangzhou Women's Hospital (Hangzhou Maternity and Child Health Care Hospital). G-band staining was applied for the preparation of the chromosome specimens. At least 30 metaphases were counted and 6 metaphases were karyotyped for each iPSC line.

Alkaline phosphatase staining

Alkaline phosphatase (ALP) staining was performed using the VECTOR Blue Alkaline Phosphatase Substrate Kit (Vector Laboratories) following the manufacturer's instructions.

Teratoma formation

Five-week-old female NOD/SCID mice were purchased (GemPharmatech). The iPSCs were suspended in 0.5 ml of mTeSR1 (at 4×10^{6} cells/100 μl). After mixing with 0.5 ml of Matrigel, the cells were injected into the armpits and back of NOD/SCID mice. Four to six weeks later, teratomas were observed, which increased to 0.5-1.5 cm in size. Teratomas were excised and fixed with 4% paraformaldehyde (PFA) (Sangon Biotech). Teratoma sections were stained with hematoxylin and eosin (H&E) and examined for three germ layers under a microscope.

Gene correction of TRPM4 T262M mutation by CRISPR/Cas9

Guide DNA (gDNA) was designed at http://crispr.mit.edu, which was cloned into lentiCRISPRv2 plasmid. Correction of c.785C>T (p.T262M) mutation in patient iPSCs via homologous recombination with single-stranded oligo deoxynucleotide (ssODN) as repair template was carried out as previously described with slight modifications. Lipofectamine 3000 was used for cell transfection. When cells were cultured and expanded to 50-80% confluence in 12-well culture plate, 125 μl Opti-Mem (Gibco) containing 1.25 μg ssODN and 1.25 μg plasmid was added. 6 hours after transfection, cells were removed from Lipofectamine 3000, passaged into 60-mm dish, and then placed in mTeSR1 with ROCK inhibitor Y-27632 (Selleck). One day after transfection, cells were placed in mTeSR1 with ROCK inhibitor Y-27632 and puromycin. After additional 2 days, puromycin-resistant clones were picked and then verified by genomic PCR and DNA sequencing (Sangon Biotech). Primer sequences used for genomic PCR are listed as followed: Forward, 5'-CACCGTCACAGCAGAAGACGGGCGT-3'; Reverse, 5'-AAACACGCCCGTCTTCTGCTGTGAC-3'. The positive iPSC clones were expanded and used for further experiments.

Cardiac differentiation

Different iPSCs were cultured and expanded until passage 20, and then differentiated into cardiomyocytes using a 2D monolayer differentiation protocol. Briefly, ~10^5 undifferentiated cells were dissociated and re-plated into matrigel-coated 6-well plates. Cells were cultured and expanded to 85% cell confluence, and then treated for 2 days

with 6 μM CHIR99021 (Axon Medchem) in RPMI 1640 (Gibco) and B27 supplement minus insulin (RPMI+B27-Insulin) (Gibco) to activate Wnt signaling pathway. On day 2, cells were placed in RPMI+B27-Insulin with CHIR99021 removal. On days 3-4, cells were treated with 5 μM IWR-1 (Sigma-Aldrich) to inhibit Wnt signaling pathway. On days 5-6, cells were removed from IWR-1 treatment and placed in RPMI+B27-Insulin. From day 7 onwards, cells were placed and cultured in RPMI 1640 (Gibco) and B27 supplement with insulin (RPMI+B27+Insulin) (Gibco) until beating was observed. Cells were glucose-starved for 3 days with RPMI+B27+Insulin for the purification. Cardiomyocytes of day 30-40 after cardiac differentiation were utilized for downstream functional assays in this study.

FACS analysis of iPSC-CMs

Monolayer iPSC-CMs were dissociated into single cells using 0.25% Tripsin-EDTA (Gibco) for 5 minutes at 37 ℃. Cells were pelleted and fixed with 4% PFA for 10 minutes on ice. Every step was washed with phosphate buffered saline (PBS) (Sangon Biotech) before sample centrifugation. Cells were stained with TNNT2 (Abcam, ab8295) at 4 ℃, and FITC-conjugated goat anti-mouse IgG antibody (Invitrogen, A11001) was used as secondary antibody.

Quantitative real-time PCR (qPCR)

Total RNA isolation from iPSC-CMs was done using RNeasy Mini Kit (Qiagen). RNA concentration was measured using UV spectrophotometry at 260 nm (Nanodrop 2000, Thermo Scientific). cDNA was obtained using the High Capacity cDNA Reverse Transcription Kit (Applied Biosystems). qPCR was performed using SYBR

Green PCR Master Mix (Takara). Primer sequences used in this study were listed in **Table S4**. Each reaction was run in triplicates or quadruplicate using an Applied Biosystems Viia7 Dx (Thermo Fisher Scientific). Gene expression values were normalized to the average expression of housekeeping gene GAPDH.

Immunofluorescent staining

Cells were fixed with 4% PFA for 15 minutes, permeabilized with 0.1% Triton X (Sangon Biotech) for 5 minutes, and blocked with 3% BSA (Sigma-Aldrich) for 1 hour. Cells were subsequently stained with appropriate primary antibodies and AlexaFluor conjugated secondary antibodies (Life Technologies). Nuclei were stained with DAPI (Roche Diagnostics). For the staining of pluripotency markers, the primary antibodies were OCT4 (Santa Cruz Biotechnology, sc-8628), NANOG (Santa Cruz Biotechnology, sc-33759), SSEA-4 (Abcam, ab16287) and SOX2 (Abcam, ab171380). For the staining of cardiac-specific markers, the primary antibodies were TNNT2 (Abcam, ab45932) and α-actinin (Abcam, ab9465). Pictures were taken with 63× objective on confocal microscope (Nikon, A1) using NIS-Elements AR software (Nikon).

Western blot

The iPSC-CMs were grown in 6-well plates to 80% confluence, detached with TrypLE (Gibco), and then pelleted at 1000 rpm for 3-5 minutes at 4 ℃. After washing with DPBS, the pellets were re-suspended in 50-100 μl lysis buffer. Lysates were placed on ice for 30 minutes and the supernatants were collected after centrifuging at 12000 rpm for 5 minutes. Protein concentration was measured using a BCA kit

(Pierce). Western blot was performed with the following antibodies: TRPM4 (Alomone Labs, ACC-044), GFP (YEASEN, 31002ES20), Na^+/K^+-ATPase (Abcam, ab7671), PDGFRB (Abcam, ab32570) and GAPDH (Abmart, M200006).

Surface expression of TRPM4 in HEK293T cells

HEK293T cells transfected with TRPM4 plasmid were washed with DPBS at pH 7.4. Membrane proteins were biotinylated by incubating cells with 2.5 mg/ml of EZ-link sulfo-NHS-LCLC-biotin (Pierce) in DPBS for 30 minutes at 4 ℃. Cells were then washed 3 times in DPBS with 100 mM glycine, then with DPBS containing 20 mM glycine, and scrapped in Triton X-100 lysis buffer (1% Triton X-100, 50 mM Tris/HCl pH 7.4, 150 mM NaCl, 1 mM EDTA and Complete Protease Inhibitor Cocktail (Roche). Lysates were obtained after 1 hour rotating at 4 ℃. Insoluble materials were removed by centrifugation. Supernatants were incubated with Ultralink Immobilised NeutrAvidin beads (Pierce) overnight at 4 ℃. The beads were precipitated and washed with Triton X-100 lysis buffer, then in saline solution (5 mM EDTA, 350 mM NaCl and 0.1% Triton X-100 in PBS pH 7.4) and finally in 10 mM Tris/HCl pH 7.4. Precipitated beads were re-suspended in SDS-PAGE loading buffer and heated for 5 minutes at 70 ℃. Proteins were resolved in 4% SDS-PAGE gels and transferred to polyvinylidene fluoride (PVDF) membranes (Millipore). Antibodies against GFP (YEASEN, 31002ES20) and GAPDH (Abmart, M200006) were used in this experiment.

TRPM4 current recordings in HEK293T cells

Human embryonic kidney 293T cells (HEK293T cells) transfected with WT or

T262M mutant TRPM4 plasmid were used for recording TRPM4 current. Membrane potential was clamped at 0 mV for both inside-out and whole cell recordings. For inside-out recordings, a 300-ms step at +80 mV was used, followed by a second 300-ms step at -80 mV. The pipette solution contained (in mM) 156 NaCl, 5 $CaCl_2$, 10 Glucose, 10 HEPES (pH 7.4) and the basic bath solution contained (in mM) 156 NaCl, 1 $MgCl_2$, 10 HEPES (pH 7.2). $CaCl_2$ was added to basic bath solution according to a calcium concentration gradient from 0.1 mM to 20 mM. Cells were washed by basic bath solution at every turn. For whole-cell recordings, the bath solution contained (in mM) 156 NaCl, 5 $CaCl_2$, 10 glucose, and 10 HEPES (pH 7.2) and the pipette solution contained (in mM) 156 CsCl, 1 $MgCl_2$, 9.87 $CaCl_2$, 10 EGTA, and 10 HEPES (pH 7.4). The current traces were elicited by voltage ramps for 250 ms from -120 to +130 mV.

Ca^{2+} imaging

The iPSC-CMs grown on coverslips were loaded with RPMI 1640 without Phenol Red (Invitrogen) supplemented with 5 μM Fura-2 AM (the stock of Fura-2 AM was pre-dissolved in 20% Pluronic F-127 solution in dimethyl sulfoxide (DMSO)) for 30 minutes in the dark at room temperature. After washing with pre-warmed DPBS and RPMI 1640 for two times, the cells were immersed in imaging buffer for 30 minutes before the experiment. Fluorescent signals obtained upon excitation at 340 nm (F340) and 380 nm (F380). Amplitude of Ca^{2+} transient is defined as the ratio of F340/F380.

Action potential recordings from iPSC-CMs

The iPSC-CMs were mechanically and enzymatically dissociated to obtain single

cells, which were seeded on Matrigel-coated glass coverslips (Warner Instruments). Cells with spontaneous beatings were selected and action potentials were recorded using an EPC-10 patch clamp amplifier (HEKA). Continuous extracellular solution perfusion was achieved using a rapid solution exchanger (Bio-logic Science Instruments). Data were acquired using PatchMaster software (HEKA) and digitized at 1 kHz. Data analyses were performed using Igor Pro (Wavemetrics) and Prism (Graphpad). A TC-344B heating system (Warner Instruments) was used to maintain the temperature at 35.5-37 ℃. Tyrodes solution was used as the external solution containing 140 mM NaCl, 5.4 mM KCl, 1 mM $MgCl_2$, 10 mM glucose, 1.8 mM $CaCl_2$ and 10 mM HEPES (pH 7.4 with NaOH at 25 ℃). The internal solution contained 120 mM KCl, 1 mM $MgCl_2$, 10 mM HEPES, 3 mM Mg-ATP, and 10 mM EGTA (pH 7.2 with KOH at 25 ℃). Key action potential parameters were quantified, including beating rate, maximal diastolic potential (MDP), overshoot, action potential amplitude (APA), action potential duration at 50%, 70% and 90% repolarization (APD_{50}, APD_{70}, APD_{90}) and maximal upstroke velocity (V_{max}). Ventricular-like myocytes were distinguished based on the action potential morphology and action potential parameters, which exhibit a clear plateau phase, larger APA and V_{max} values, more negative MDP values, $APD_{30–40}/APD_{70–80} > 1.5$ and $APD_{90}/APD_{50} \leq 1.3$.

Endogenous Ca^{2+} current recordings from iPSC-CMs

Endogenous Ca^{2+} currents were isolated from single iPSC-CMs using the ruptured patch clamp technique with conventional voltage clamp protocols. Bath solution contained: 160 mM TEA-Cl, 5 mM $CaCl_2$, 1 mM $MgCl_2$, 10 mM glucose, 10 mM

HEPES, 0.01 mM TTX, 2 mM 4-AP (pH 7.4 with CsOH). Pipette solutions contained: 145 mM CsCl, 5 mM NaCl, 1 mM $CaCl_2$, 5 mM MgATP, 5 mM EGTA, and 10 mM HEPES (pH 7.2 with CsOH). All currents were normalized to cell capacitance to obtain current density. Steady-state activation and inactivation curves were fitted by using a Boltzmann equation: $f = 1/\{1 + \exp[\pm(V - V_{1/2})/k]\}$, in which $V_{1/2}$ is half-maximum (in)activation potential and k is slope factor.

RNA-Sequencing

The sequencing data was filtered with SOAPnuke (v1.5.2) by (1) Removing reads containing sequencing adapter; (2) Removing reads whose low-quality base ratio (base quality less than or equal to 5) is more than 20%; (3) Removing reads whose unknown base ('N' base) ratio is more than 5%, afterwards clean reads were obtained and stored in FASTQ format. The clean reads were mapped to the reference genome using HISAT2 (v2.0.4). Bowtie2 (v2.2.5) was applied to align the clean reads to the reference coding gene set, then expression level of gene was calculated by RSEM (v1.2.12). The heatmap was drawn by pheatmap (v1.0.8) according to the gene expression in different samples. Essentially, differential expression analysis was performed using the DESeq2 (v1.4.5) with Q value≤ 0.05. To take insight to the change of phenotype, GO (http://www.geneontology.org/) and KEGG (https://www.kegg.jp/) enrichment analysis ofannotated different expressed gene was performed by Phyper (https://en.wikipedia.org/wiki/Hypergeometric_distribution) based on Hypergeometric test. The significant levels of terms and pathways were corrected by Q value with a rigorous threshold (Q value≤ 0.05) by Bonferroni.

Molecular modeling

The structure of the hTRPM4 was acquired from Protein Data Bank (6BQR), and the structure was relaxed by Rosetta relax application, models with lowest energy scores were chosen as template. Backrub application was used to create T262M point mutation. The backrub algorithm rotates local segments of the protein backbone as a rigid body about an axis defined by the starting and ending atoms of the segment. The updated atoms branching of the main chain at the pivot points minimized the bond angle strain. These moves are accepted or rejected using the Metropolis criterion. The application takes as input the crystal structure hTRPM4 of the wild-type (which must be relaxed), and generates 60 structural models of the point-mutant, we chose the lowest energy scores as the result.

Commands in Rosetta to perform relax:

-database /home/xlz/rosetta_2015.25/main/database

-ignore_unrecognized_res

-in:file:fullatom

-in:file:s /home/xlz/Desktop/hTRPM4.pdb

-relax:fast true

-relax:constrain_relax_to_start_coords

-relax:coord_constrain_sidechains

-relax:ramp_constraints false

-relax:jump_move true

-relax:default_repeats 1

-nstruct 100

-out:file:fullatom

-out:file:silent /home/xlz/Desktop/hTRPM4_relaxed.silent

-out:file:silent_struct_type binary

-out:output

-overwrite

Commands in Rosetta to perform mutation:

-database /home/xlz/rosetta_2015.25/main/database

-in:file:fullatom

-ignore_unrecognized_res

-s /home/xlz/Desktop/hTRPM4.pdb

-backrub:ntrials 1000

-nstruct 60

-mc_kt 0.6

-resfile /home/xlz/Desktop/mutation.resfile

-out:file:silent /home/xlz/Desktop/hTRPM4_T291.silent

-out:file:silent_struct_type binary

-overwrite

mutation.resfile:

NATRO

start

291 A PIKAA M

291 B PIKAA M

291 C PIKAA M

291 D PIKAA M

PDGFRB knockdown

Lipofectamine RNAiMax (Life Technologies) was used for gene knockdown experiments according to the manufacturer's instructions. Scrambled and siRNA against PDGFRB were designed by GenePharma. Patient iPSC-CMs were transfected with either scrambled or PDGFRB siRNA (40 nM) for 48 hours before downstream experiments.

Compounds and solutions

All the chemicals used in the electrophysiological experiments were purchased from Sigma-Aldrich. Verapamil was purchased from Sigma and stock solutions were prepared as 1 mM in H_2O. Crenolanib and Sunitinib were both purchased from Selleck and stock solutions were prepared as 1 mM and 5 mM in DMSO, respectively. MG132 and chloroquine were both purchased from Targetmol and stock solutions were prepared as 10 mM and 20 mM in DMSO, respectively.

Data availability

The accession number for the RNA-Seq data reported in this study is PRJNA691108.

Statistical analysis

Statistical significance was determined by unpaired two-tailed Student's t-test to compare two groups and by One-way ANOVA to compare multiple groups. A *p* value of < 0.05 was considered statistical significant. Data were shown as mean ± sem and

analyzed by GraphPad Prism 8 (GraphPad Software).

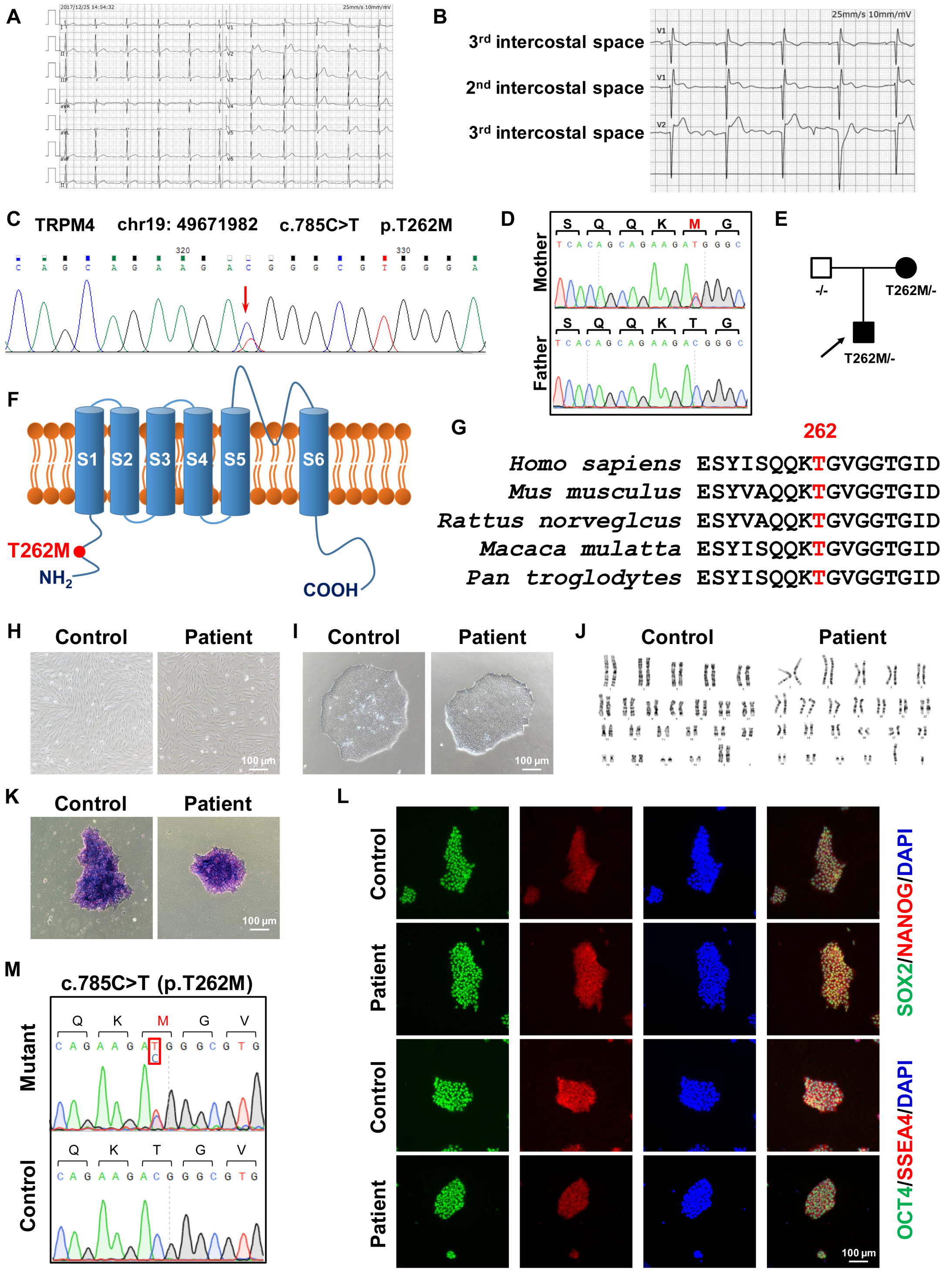

Figure S1. Generation and characterization of patient-specific iPSCs carrying TRPM4 T262M. A. Typical electrocardiogram (ECG) from the recruited patient demonstrating Brugada pattern. **B.** Recording leads V1 to V2 from one or two intercostals spaces higher than their standard positions (i.e. 3rd or 2nd intercostals space for lead V1 and V2). **C.** Genetic testing identifying missense mutation of T262M in *TRPM4* gene carried by the patient. **D.** Verification of the T262M mutation in the patient's mother and father. The mutation is absent in the patient's father but

present in his mother. **E.** The pedigree of the BrS family. The arrow indicates the proband. Males are denoted by squares and females are denoted by circles. Solid symbols indicate presence of T262M mutation. **F.** Schematic representation of the TRPM4 channel. The T262M mutation locates at the N terminus of TRPM4 indicated by the red arrow. **G.** Sequence alignment of amino acids adjacent to Thr262 in TRPM4 gene among species. The silico analysis of the missense mutation was predicted to be deleterious consistently by PolyPhen-2 and Sorting Intolerant From Tolerant (SIFT). Moreover, the mutation is absent in the Human Gene Mutation Database (HGMD) and ClinVar, and it was not found in the 1000 Genomes data set (1000G) or gnomAD database. Accordance with the American College of Medical Genetics and Genomics (ACMG) guidelines, this mutation was graded as "variant of uncertain significance (VUS)". **H.** Typical morphology of skin fibroblasts from the healthy control subject and the patient carrying TRPM4 T262M. The healthy control subject recruited in the study was a 21-year-old female with normal ECG and no personal or family history of syncope or sudden cardiac death (SCD). Scale bar, 100 μm. **I.** Typical morphology of control and patient iPSCs. Scale bar, 100 μm. **J.** Representative graphs of karyotype of control and patient iPSCs. **K.** Representative graphs of ALP staining of control and patient iPSCs. Scale bar, 100 μm. **L.** Representative graphs of pluripotent staining of control and patient iPSCs using SOX2 (green), NANOG (red), OCT4 (green) and SSEA4 (red). DAPI indicates nuclear staining (blue). Scale bar, 100 μm. **M.** Confirmation of the existence of *TRPM4* T262M mutation in patient iPSCs but not in control iPSCs.

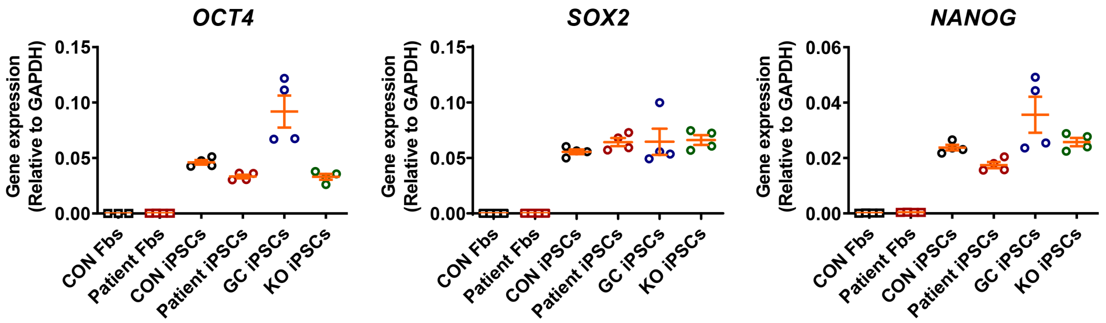

Figure S2. Comparison of the pluripotency gene expression between fibroblasts and iPSCs. Bar graph to compare the mRNA expression of the pluripotency genes between skin fibroblasts (Fbs) and iPSCs by qPCR, including *OCT4*, *SOX2* and *NANOG*.

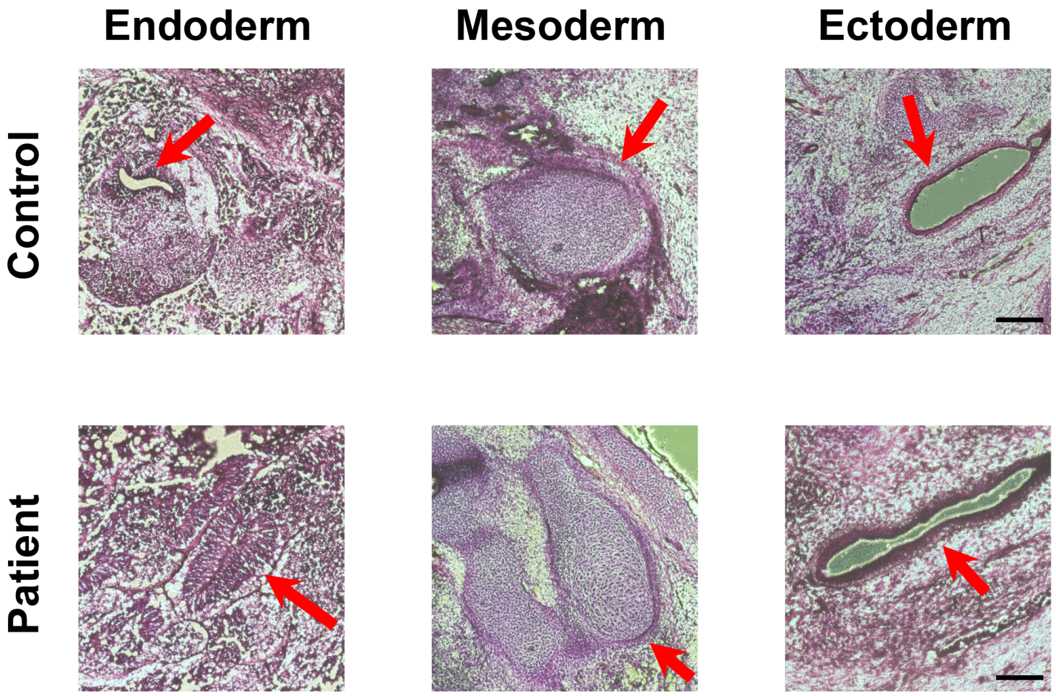

Figure S3. Teratoma formation assay. Teratoma formation assay using control and patient-specific iPSCs showing derivations of three embryonic germ layers (endoderm, mesoderm and ectoderm). Scale bar, 200 μm.

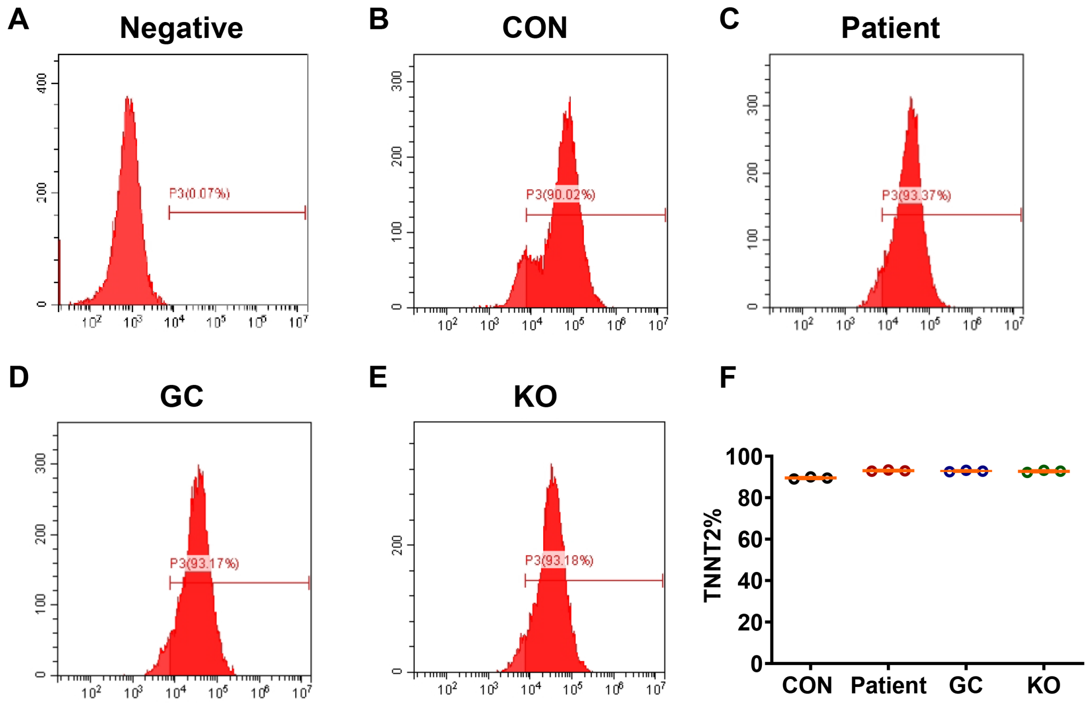

Figure S4. Analysis of cardiac differentiation efficiency by FACS. A-E. FACS analysis of TNNT2-positive cells in negative control, control (CON), patient-specific (Patient), gene-corrected (GC) and TRPM4 KO (KO) iPSC-CMs. **F.** Bar graph to compare the percentage of TNNT2-positive cells between different groups.

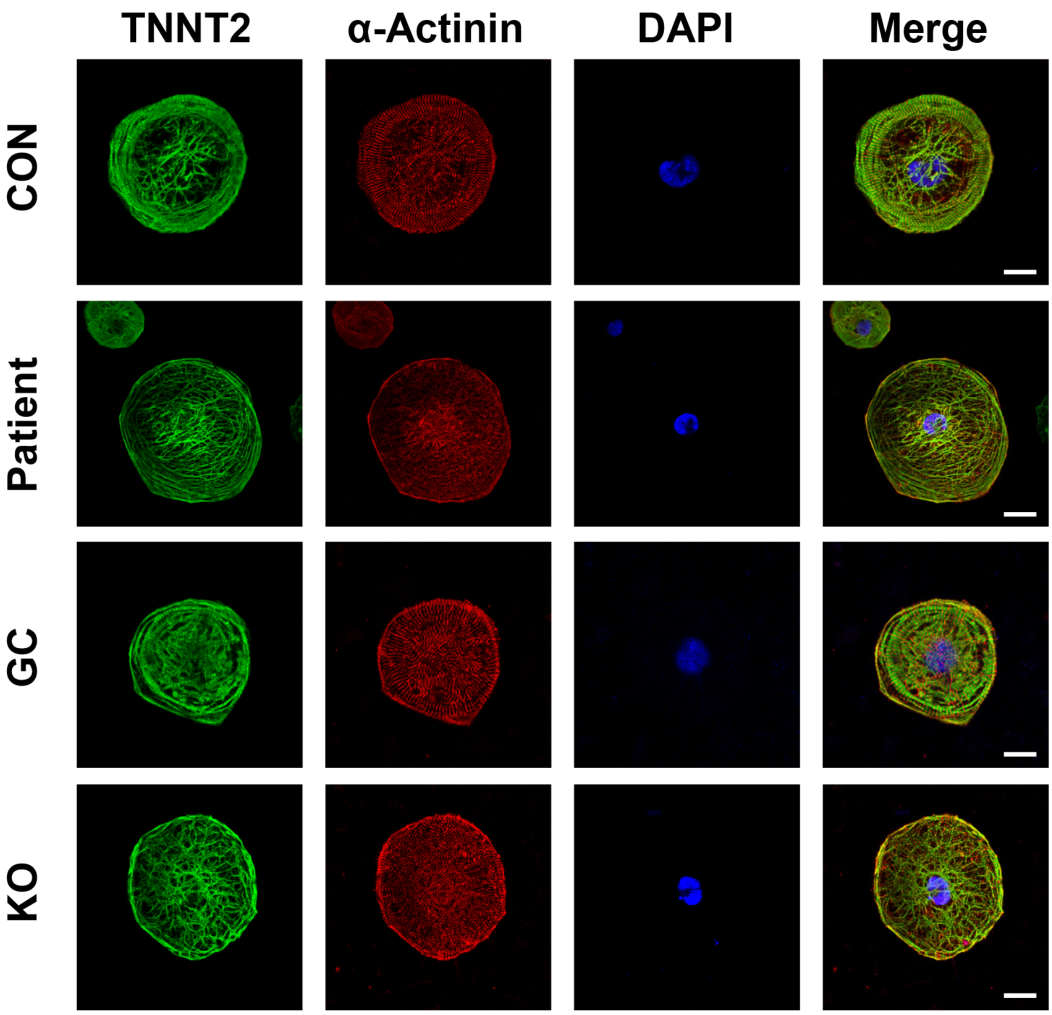

Figure S5. Immunofluorescent staining of cardiac-specific markers in iPSC-CMs. Representative graphs of cardiac-specific staining by TNNT2 (green) and α-actinin (red) in CON, Patient, GC and KO iPSC-CMs. The morphology and shape was indistinguishable between different groups. DAPI indicates nuclear staining (blue). Scale bar, 20 µm.

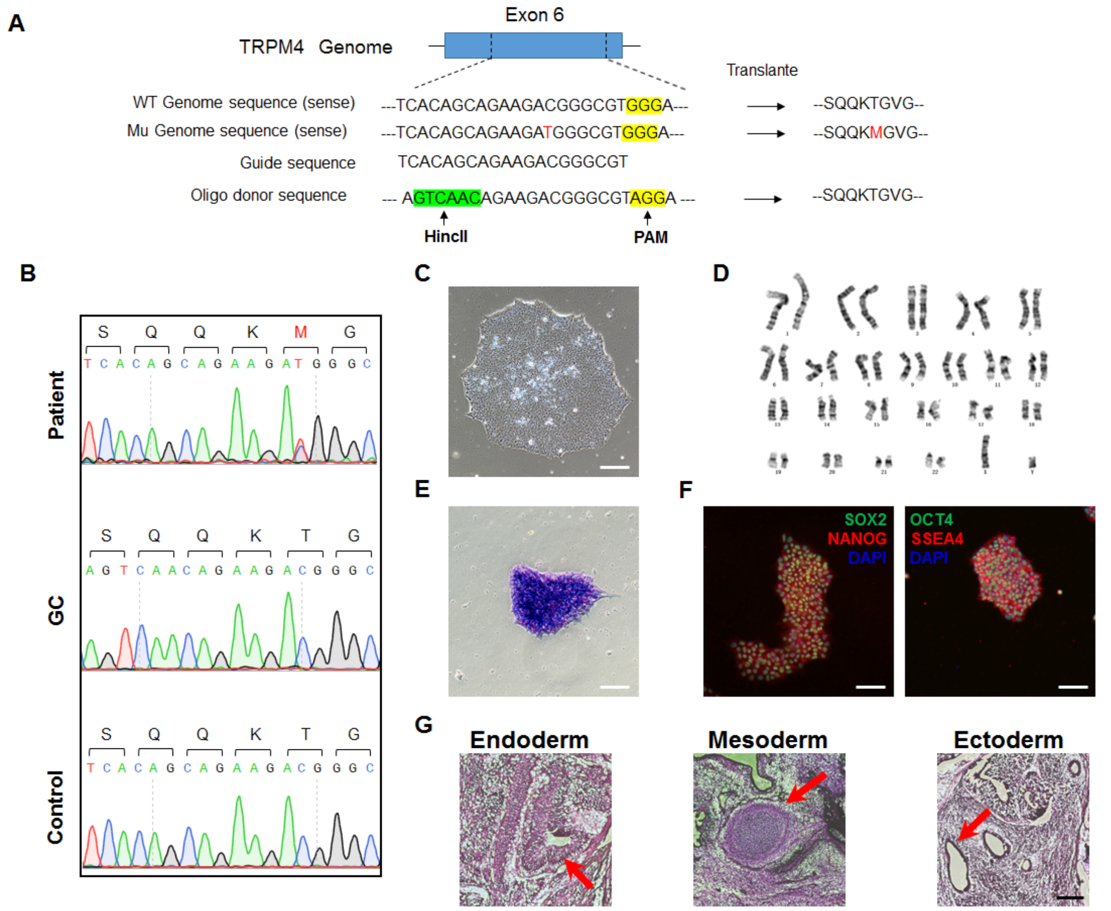

Figure S6. Gene correction of TRPM4 T262M by CRISPR/Cas9 at the iPSC level. **A.** Strategy of correcting TRPM4 c.785 C>T (p.T262M) mutation. The mutation in DNA and amino acid of patient specific iPSC is shown in red, the NGG PAM sequence is shown in yellow and the designed restriction sites are shown in green. **B.** DNA sequencing demonstrates the correction of c.785 C>T (p.T262M) mutation. The mutant amino acid sequence in patient specific iPSC is shown in red. Using CRISPR/Cas9 system and a donor plasmid containing WT *TRPM4* sequence and homology arms (HA) as the repair template, we successfully obtained 3 positive iPSC clones (clone#4, 24 and 32), in which correction of specific TRPM4 T262M mutation was confirmed by genomic sequencing. **C.** Typical morphology of gene-corrected (GC) iPSCs. Scale bar, 100 μm. **D.** Representative graphs of karyotype of GC iPSCs. **E.** Representative graphs of alkaline phosphatase (ALP) staining of GC iPSCs. Scale bar, 100 μm. **F.** Representative graphs of pluripotent staining of control and patient iPSCs using SOX2 (green), NANOG (red), OCT4 (green) and SSEA4 (red). DAPI indicates nuclear staining (blue). Scale bar, 100 μm. **G.** Teratoma formation assay using GC iPSCs showing derivations of three embryonic germ layers. Scale bar, 200 μm.

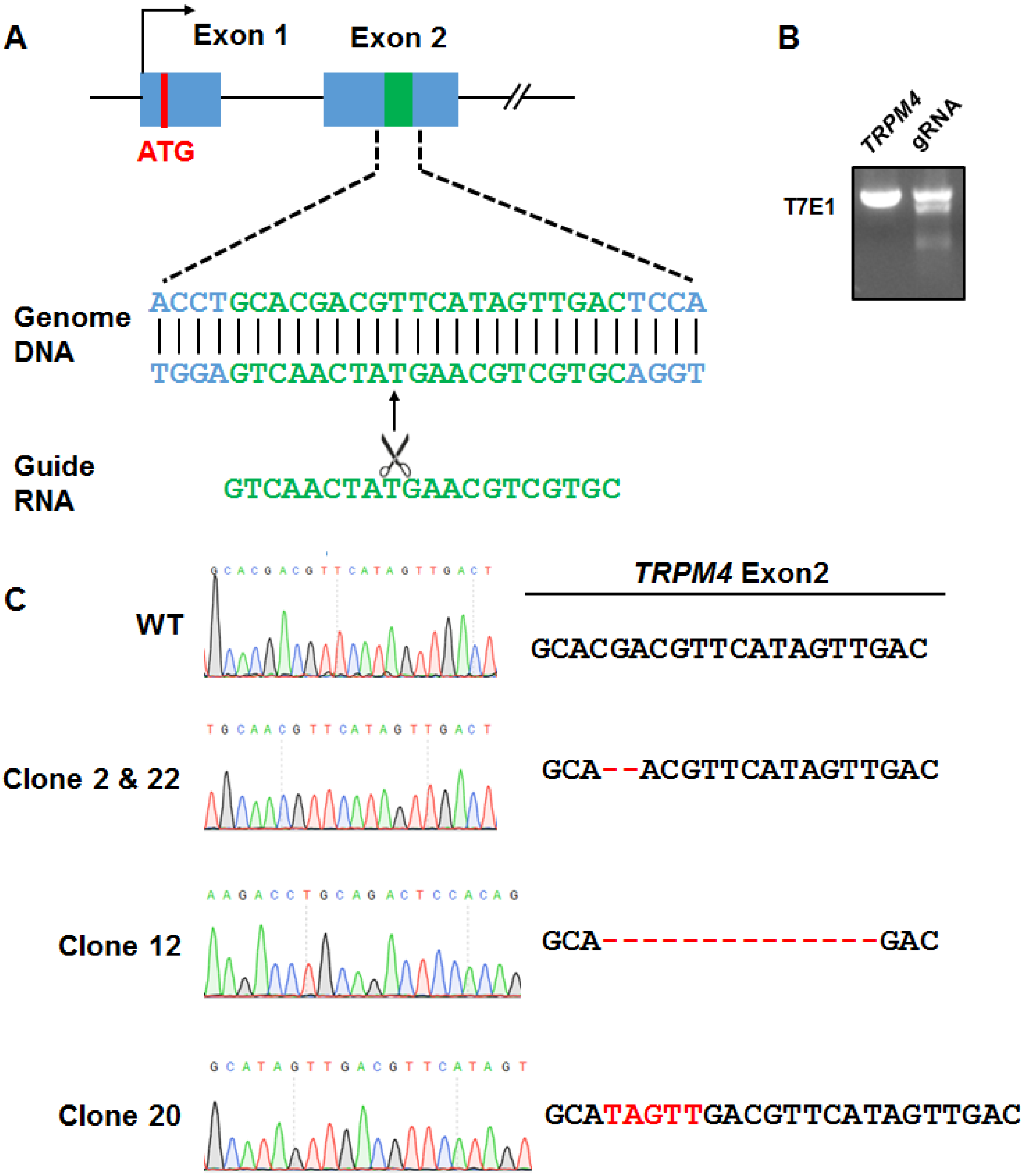

Figure S7. Generation of TRPM4 Knockout iPSC lines by CRISPR/Cas9. A. Schematic of Cas9/gRNA-targeting sites in TRPM4. TRPM4 knockout (KO) iPSC lines were generated by targeting exon 2 of the *TRPM4* gene locus using CRISPR/Cas9. Aforementioned control iPSCs were transfected with a plasmid encoding the TRPM4-targeting guide RNA (gRNA) and single colonies were subsequently picked and genotyped. Four (clone#2, 12, 20 and 22) out of 22 colonies were identified to contain homozygous nonsense mutation. **B.** TRPM4-specific sRNA RNP-mediated mutations measured by the T7E1 assay. **C.** Sanger sequencing of WT iPSCs, TRPM4 knockout (KO) Clone#2, #12, #20 and #22, respectively.

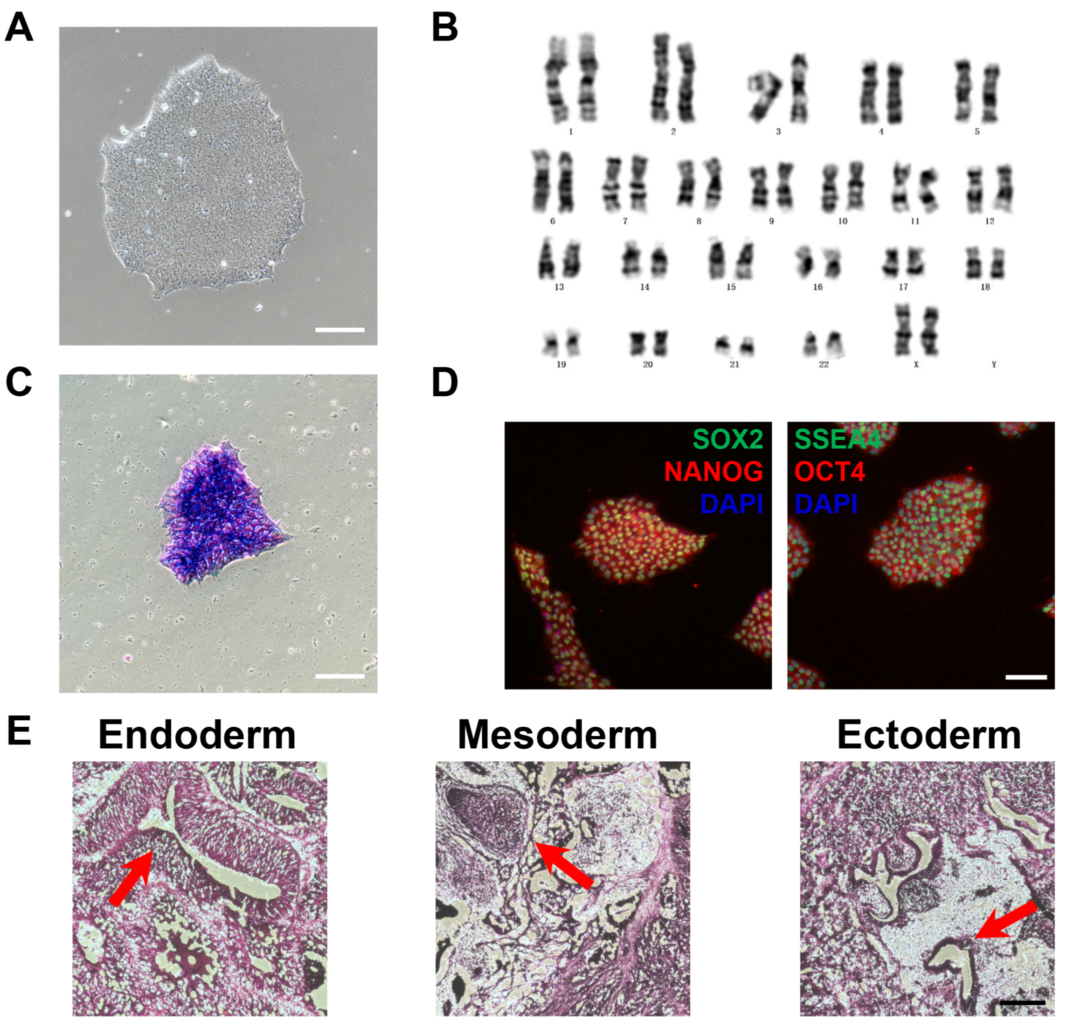

Figure S8. Characterization of the generated TRPM4 KO iPSCs. A. Typical morphology of TRPM4 KO iPSCs. Scale bar, 100 μm. **B.** Representative graph of karyotype of TRPM4 KO iPSCs. **C.** Representative graph of ALP staining of TRPM4 KO iPSCs. Scale bar, 100 μm. **D.** Representative graphs of pluripotent staining of TRPM4 KO iPSCs using SOX2 (green), NANOG (red), SSEA4 (green) and OCT4 (red). DAPI indicates nuclear staining (blue). Scale bar, 100 μm. **E.** Teratoma formation assay using TRPM4 KO iPSCs showing derivations of three embryonic germ layers. Scale bar, 200 μm.

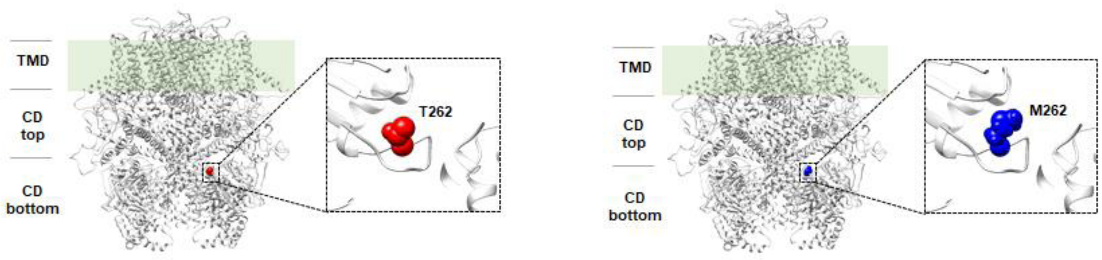

Figure S9. *In silico* modeling of WT and T262M TRPM4 channels. Localization of Thr262 (red) and Met262 (blue) in the human TRPM4 cryo-EM structure (PDB entry code 6BQR). TMD stands for transmembrane channel domain and CD stands for cytoplasmic domain.

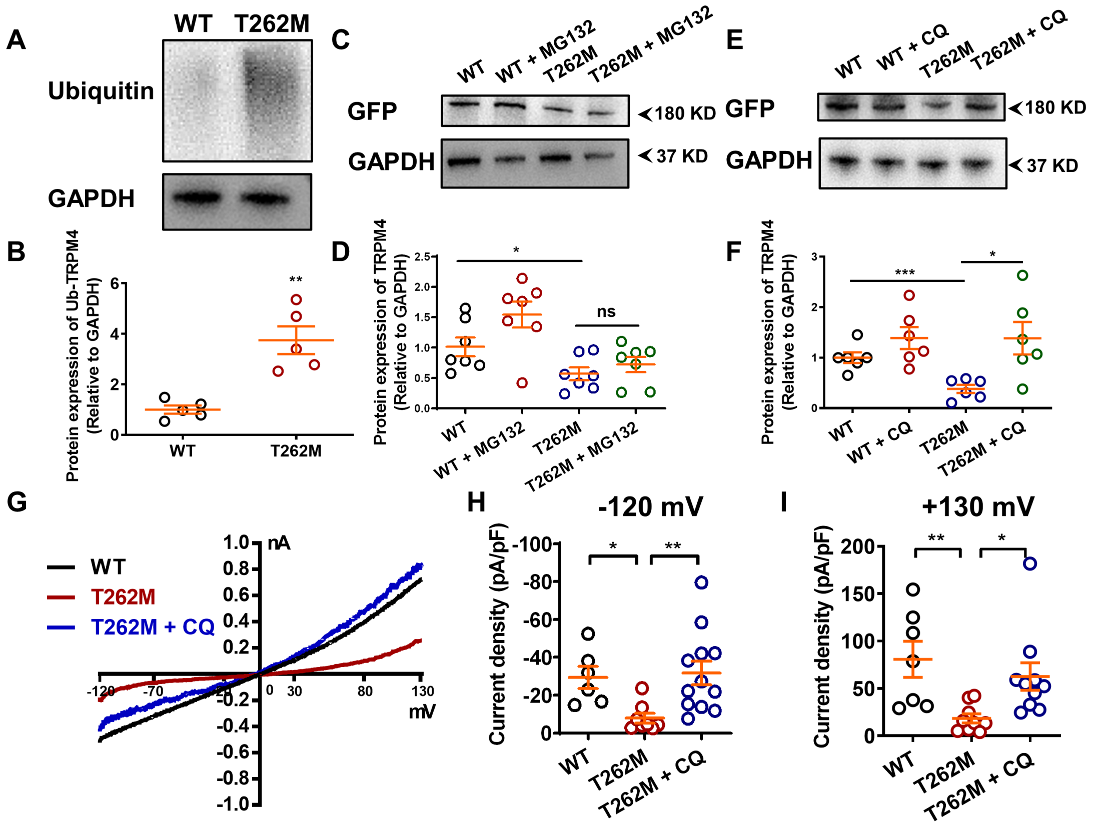

Figure S10. T262M results in TRPM4 protein degradation by ubiquitin in the lysosome. A. Western blot analysis of protein expression of ubiquitinated TRPM4 (Ub-TRPM4). To determine if the down-regulated expression of TRPM4 protein is degraded by ubiquitin, Western blots were performed to detect the ubiquitination level of TRPM4 in HEK293 cells expressing both WT and T262M. **B.** Scatter dot plot to compare the protein expression of Ub-TRPM4 between the two groups by unpaired two-tailed Student's t-test. n= 5 independent experiments. $^{**}P< 0.01$. **C.** Western blot analysis of total protein expression of TRPM4 in HEK293T overexpressing WT or T262M treated with dimethyl sulfoxide (DMSO) or MG132, respectively. **D.** Scatter dot plot to compare the total protein expression of TRPM4 between different groups by unpaired two-tailed Student's t-test. Inhibition of proteasome pathway by MG132 had no rescuing effect on the T262M-induced down-regulation of TRPM4 protein. n= 7 independent experiments. $^{*}P< 0.05$. **E.** Western blot analysis of total protein expression of TRPM4 in HEK293T overexpressing WT or T262M treated with DMSO or chloroquine (CQ). **F.** Scatter dot plot to compare the total protein expression of TRPM4 between different groups by unpaired two-tailed Student's t-test. n= 6 independent experiments. $^{*}P< 0.05$ and $^{***}P< 0.001$. **G.** IV curves of whole-cell TRPM4 currents recorded from HEK293T overexpressing WT, T262M treated with DMSO, or T262M treated with CQ. **H-I.** Scatter dot plots to compare the peak TRPM4 current density at -120 mV and +130 mV between different groups by One-way ANOVA (Tukey method). n= 6-12 patches. $^{*}P< 0.05$ and $^{**}P< 0.01$.

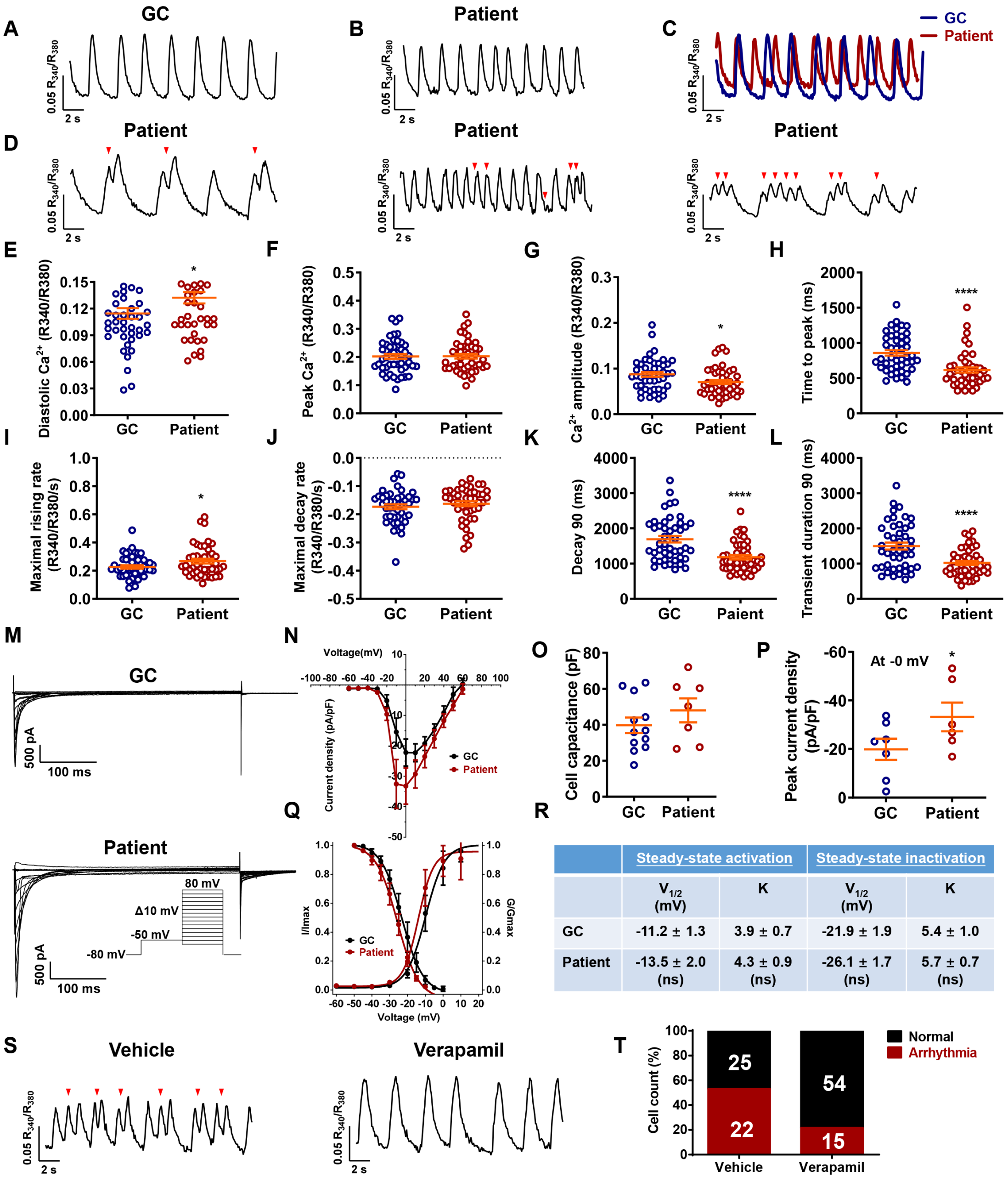

	Steady-state activation		Steady-state inactivation	
	$V_{1/2}$ (mV)	K	$V_{1/2}$ (mV)	K
GC	-11.2 ± 1.3	3.9 ± 0.7	-21.9 ± 1.9	5.4 ± 1.0
Patient	-13.5 ± 2.0 (ns)	4.3 ± 0.9 (ns)	-26.1 ± 1.7 (ns)	5.7 ± 0.7 (ns)

Figure S11. TRPM4 T262M leads to dysregulation of Ca^{2+} cycling and elevation of diastolic $[Ca^{2+}]_i$. A-B. Representative Ca^{2+} transient tracings from GC and patient iPSC-CMs. **C.** Alignment of Ca^{2+} transient tracings of GC and patient iPSC-CMs. **D.** Representative Ca^{2+} transient tracings demonstrating irregular arrhythmia-like events in patient iPSC-CMs. **E-L.** Scatter dot plots to compare key Ca^{2+} transient parameters between GC and patient iPSC-CMs by unpaired two-tailed Student's t-test. n= 45-46 in two lines. $^{*}P< 0.05$ and $^{****}P< 0.0001$. Notably, we found that diastolic $[Ca^{2+}]_i$ was higher (15.5% increase in Fura-2 ratio) in iPSC-CMs carrying the T262M mutation as compared to GC cells. Moreover, we observed significantly decreased Ca^{2+} transient amplitude, decreased time to peak, reduced maximal rising rate, decreased decay 90 and transient duration in patient iPSC-CMs in comparison to GC cells, whereas peak

Ca^{2+} transient and maximal decay rate did not show any detectable change. **M.** Representative Ca^{2+} current tracings isolated from GC and patient iPSC-CMs. Inset showing the voltage protocol. **N.** Comparison of Ca^{2+} IV curve between GC and patient iPSC-CMs. n= 6-8 in two lines. **O.** Scatter dot plot to compare the cell capacitance between GC and patient iPSC-CMs by unpaired two-tailed Student's t-test. n= 7-12 in two lines. **P.** Scatter dot plot to compare the peak Ca^{2+} current density at 0 mV between GC and patient iPSC-CMs by unpaired two-tailed Student's t-test. n= 6-7 in two lines. $^{*}P< 0.05$. **Q.** Comparison of steady-state activation and inactivation of Ca^{2+} currents between GC and patient iPSC-CMs. n= 6-8 in two lines. **R.** Table to compare key parameters of steady-state activation and inactivation between GC and patient iPSC-CMs. No significant changes in gating kinetics were observed between GC and patient iPSC-CMs. **S.** Representative Ca^{2+} transient tracings of patient iPSC-CMs before and after verapamil application (L-type Ca^{2+} channel blocker, 100 nM, 20 days). **T.** Bar graph to compare percentage of cells exhibiting regular and irregular Ca^{2+} transient pattern before and after verapamil application in patient iPSC-CMs. n= 47-69 in two lines.

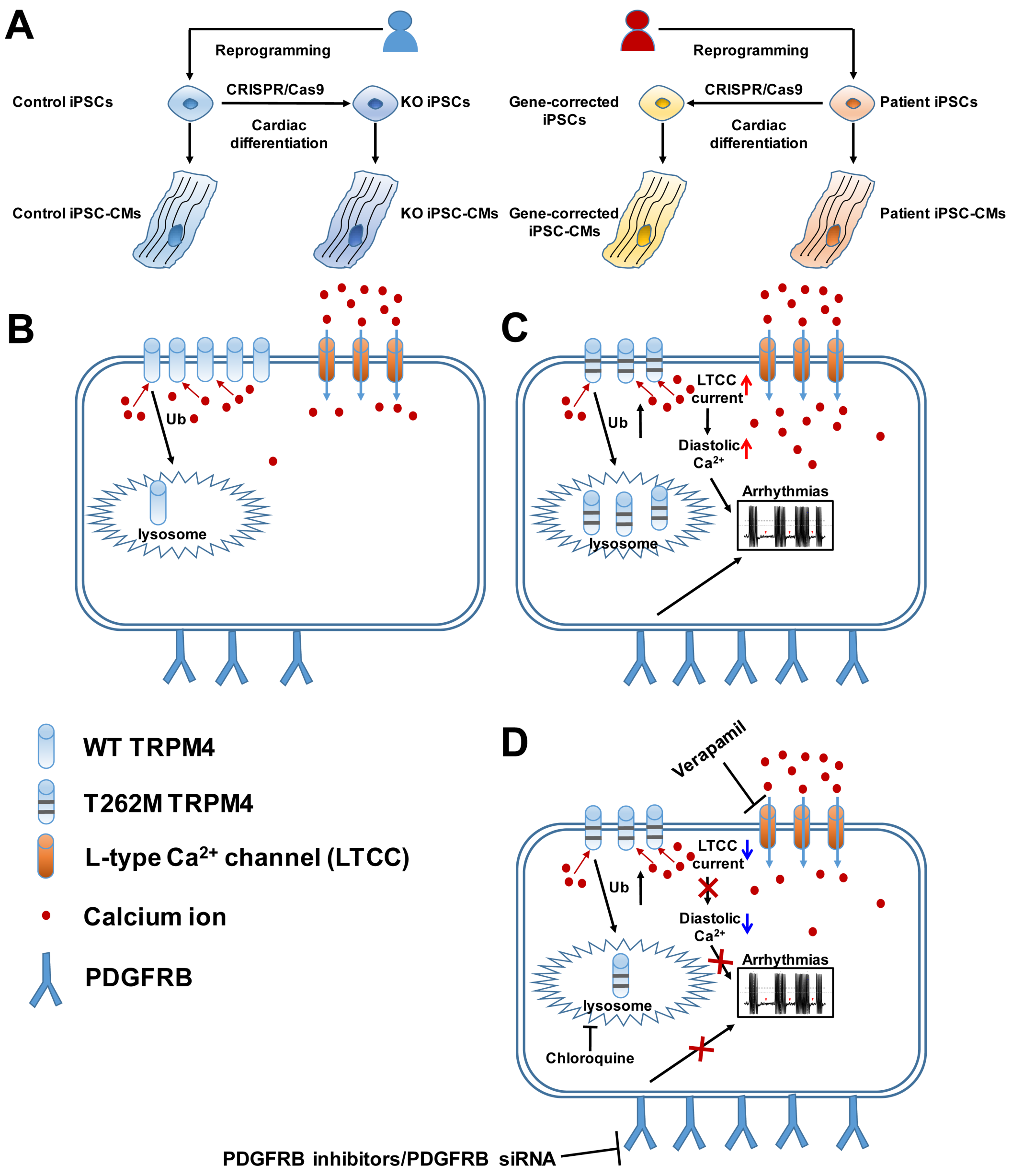

Figure S12. Disease-in-a-dish model uncovers new insights into the cause of BrS. A-D. Patient-specific iPSC-CMs generated from a VUS carrier (TRPM4 T262M) of BrS exhibited arrhythmic phenotypes, which were rescued by correcting the causal mutation. T262M conferred impaired TRPM4 channel function by enhanced ubiquitination for protein degradation via lysosomal pathway, resulting in abnormal Ca^{2+} cycling and elevated diastolic $[Ca^{2+}]_i$. Hyperactivation of PDGFRB signaling derived arrhythmic events in T262M myocytes, whereas pharmacological and genetic inhibition of PDGFRB effectively restored diastolic $[Ca^{2+}]_i$ and rescued arrhythmic phenotypes.

Table S1. Summary of action potential parameters in control, patient, GC and KO iPSC-CMs

	Beating Rate (Beats/min)	MDP (mV)	Overshoot (mV)	APA (mV)	APD_{50} (ms)	APD_{70} (ms)	APD_{90} (ms)	V_{max} (V/s)
**CON**	46 ± 3	-62.8 ± 0.7	45.2 ± 1.0	108.0 ± 1.3	375.7 ± 14.7	412.3 ± 15.5	444.3 ± 16.2	9.4 ± 0.4
**Patient**	90 ± 6	-56.2 ± 1.2	44.0 ± 1.4	100.1 ± 0.8	244.3 ± 10.7	269.1 ± 11.5	290.6 ± 11.9	11.4 ± 0.7
**GC**	63 ± 5	-53.8 ± 0.8	46.3 ± 1.1	99.8 ± 1.5	331.1 ± 25.9	361.6 ± 27.1	386.4 ± 27.8	12.2 ± 0.5
**KO**	63 ± 10	-57.2 ± 1.3	40.6 ± 1.7	97.1 ± 2.0	262.5 ± 25.0	299.5 ± 29.4	325.1 ± 31.3	10.0 ± 0.7

Table S2. Summary of Ca^{2+} transient parameters in patient and GC iPSC-CMs

	Diastolic Ca^{2+} (R340/R380)	Peak Ca^{2+} (R340/R380)	Ca^{2+} amplitude (R340/R380)	Time to peak (ms)	Maximal rising rate (R340/R380/s)	Maximal decay rate (R340/R380/s)	Decay 90 (ms)	Transient duration 90 (ms)
**Patient**	0.132 ± 0.006	0.203 ± 0.008	0.070 ± 0.005	615.50 ± 36.96	23.2 ± 1.1	0.268 ± 0.016	1181.0 ± 62.8	1022.0 ± 58.2
**GC**	0.115 ± 0.006	0.202 ± 0.009	0.087 ± 0.005	858.30 ± 39.36	17.9 ± 0.7	0.226 ± 0.012	1693.0 ± 92.6	1499.0 ± 98.8

Table S3. Summary of iPSC lines in this study

	Age	Gender	Ethnicity	Somatic cells	Reprogramming method	iPSC lines established
**CON**	21	Female	Han Chinese	Skin fibroblasts	Sendai virus	Clone #2, Clone #7
**Patient**	22	Male	Han Chinese	Skin fibroblasts	Sendai virus	Clone #2, Clone #7

Table S4. Primers used for qPCR in this study

Genes	Forward	Reverse
PDGFRB	TGTGAATGACCATCAGGATGAA	CAGCTCAGCAAATTGTAGTGTG
OCT4	CCTCACTTCACTGCACTGTA	CAGGTTTTCTTTCCCTAGCT
SOX2	CCCAGCAGACTTCACATGT	CCTCCCATTTCCCTCGTTTT
NANOG	CATGAGTGTGGATCCAGCTTG	CCTGAATAAGCAGATCCATGG
GAPDH	GGTCGGAGTCAACGGATTTG	CGGTGCCATGGAATTTGCC
